# Peanut allergens: new consolidated findings on structure, characteristics, and allergome 

**DOI:** 10.5414/ALX01418E

**Published:** 2018-09-01

**Authors:** W.M. Becker, A. Petersen, U. Jappe

**Affiliations:** 1 Klinische und Molekulare Allergologie, Klinische Pneumologie des Forschungszentrums Borstel,; 2 Universitäts-Hautklinik Lübeck

**Keywords:** Ara h 1, Ara h 2, Ara h 3, Ara h 4, Ara h 5, Ara h 6, Ara h 7, Ara h 8, Ara h 9, Ara h 10, Ara h 11, peanut allergens, PNA, peanut oleosin, peanut LTP

## Abstract

Immunoglobulin E-mediated food allergy is the result of a complex pathomechanism. Factors contributing to the dysfunction of the immune system are the allergenic sources and the variable matrix effects arising from the processes involved in interaction with the gastrointestinal tract, the allergens themselves through their structural features, and the specific behavior of the individual immune system. The starting point for elucidating the pathomechanism of food allergy is the identification of allergens and the description of their structure. They are the basis for in vitro diagnostics as well as the development of immunotherapeutic drugs. With regard to Class I food allergy, peanut allergy affects by far the largest group of patients. 11 allergens have been identified in peanuts. Ara h 1, Ara h 3, and Ara h 4 belong to the cupin superfamily, Ara h 2, Ara h 6, and Ara h 7 to the prolamin superfamily; Ara h 5 (profilins) and Ara h 8 (superfamily of Bet v 1-homologous proteins) are associated with aeroallergens. Peanut lipid transfer proteins (LTP) and two peanut oleosins are listed as Ara h 9, Ara h 10, and Ara h 11 by the IUIS Allergen Nomenclature Subcommittee. Peanut agglutinin (PNA) and a third oleosin have been shown to possess allergenic properties. The effect of the above specified allergens has to be considered in the context of their matrix, which is influenced by processing factors.

**German version published in Allergologie, Vol. 34, No. 8/2011, pp. 398-411.**

## Introduction 

The peanut (*Arachis hypogaea*) is one of 55 species in the genus Arachis which belongs to the legume familiy. Thus, the peanut is not a nut, but a legume like pea or lupine. Peanuts extist in two subspecies: *Arachis hypogaea hypogaea* and *Arachis hypogaea fastigata* (http://www.uniprot.org/taxonomy/3818). Both are consumed as a food and their immunologic difference is only marginal. Arachis duranensis (Ara d) and Arachis ipaensis (Ara i) are important gene sources in peanut research. Allergens cloned in homology with Arachis hypogaea are over 90% identical and are only important for in silico investigation. 

Among persistent food allergies, peanut allergy is probably the most frequent one. Its symptoms are highly variable, ranging from mild oral allergy syndrom (OAS) to anaphylactic shock, and can affect various organs. Even 4 mg of peanut can cause clear symptoms [25]. The sources of peanut allergens are also manifold and range from a slightly roasted whole peanut to a highly processed product in a snack. 

## Nomenclature of allergens 

Official allergen nomenclature uses the Latin name of the allergen source to describe single allergens. Thus, for the peanut allergens the first three letters of the genus, Ara*chis,* followed by the first letter of the species name, *h* for h*ypogaea*, and then the allergen number in the order of discovery are used, i.e., Ara h 1 for the first described peanut allergen [7, 26]. Newly detected allergens are submitted to the Allergen Nomenclature Subcommittee of the International Union of Immunologica Societies (IUIS) and registered according to the standing orders (http://www.allergen.org/) [7]. 

## Classification of food allergies 

Food allergies can be categorized into two classes [11]. This classification is based on the underlying immunologic mechanisms, the triggering allergens, and – with some reservations – on the clinical picture. 

## Class I food allergies 

According to current knowledge, sensitization in Class I food allergies takes place in the gastrointestinal tract. The triggering allergens are characterized by resistance to digestion and stability against denaturation [7]. Sensitization outside the intestine can however not be excluded [1]. The IgE reactivity is directed against stable conformational and/or sequential epitopes of these allergens. In the case of peanut allergens, 23 linear epitopes are described for Ara h 1, 10 for Ara h 2, and 4 for Ara h 3 [7, 47, 55, 56]. Allergens causing Class I food allergies are mainly found in sources like hen’s egg, cow’s milk, fish, crustaceans (shrimps), tree nuts, and legumes like soybean, lupine, and peanut. Class I food allergies mainly manifest in children and resolve with maturation of the gastrointestinal immune system. In only 20% of patients with peanut allergy does the disease resolve with age [43]. Class I food allergies can be associated with mild clinical symptoms (like oral allergy syndrome (OAS)) to severe clinical symptoms (systemic reactions like urticaria, asthma, gastrointestinal disorders) that can lead to anaphylactic shock. 

## Class II food allergies 

Class II food allergies are mainly present in adults who have already developed a prior allergy against inhalant allergens. The immunological basis for the reactivity with allergens that can cause Class II food allergies is probably their cross-reactivity with inhalant allergens [7]. In northern European areas with a high occurrence of birch trees, the phenomenon of cross-reactions is very common and usually has a clinically mild course, i.e., most patients develop an oral allergy syndrome. The most common cross-reations are birch pollen/apple/nut allergy, birch pollen/celery/spices allergy, and latex/fruit allergy. The IgE reactivity is directed against conformational epitopes that are unstable against digestion and heating. Therefore, patients suffering from a Class II food allergy like apple or hazelnut allergy tolerate apples in applesauce or hazelnut in pastries [7]. Patients with birch pollen-associated celery allergy react to the heat-instable components, patients with mugwort-associated celery allergy, on the other hand, react to the heat-stable epitopes [29]. 

## Allergen families 

Vegetable food allergens can be classified into the following major protein families: prolamins (also containing the group of non-specific lipid transfer proteins (nsLTP)), the Bet v 1 superfamily, profilins, cupins [12], and cross-reactive carbohydrate epitopes. 

### The prolamin superfamily

Prolamins are a group of storage proteins that are mainly found in the seeds of cereals and other members of the grass family. 2S-albumins and the non-specific lipid transfer proteins [12], the latter for example Ara h 9 from peanut, are proteins related to prolamins. Peanut allergens (Arachis hypogaea) Ara h 2 and Ara h 6 belong to the conglutin type of 2S-albumins. Currently they are considered the most important peanut allergens [12]. 

### The Bet v 1 superfamily

Bet v 1, the birch pollen major allergen, is one of the common vegetable pathogenesis-related proteins, PR10. The detection of its molecular structure allowed for the identification of structurally related proteins in other allergen sources. In this way, a superfamily was defined and the Bet v 1 family is only one of its members [12]. The Bet v 1-homologous allergen of the peanut is Ara h 8 [44]. 

### Profilins

Profilins are present in almost all eukaryotic cells. They are conserved proteins with a molecular weight between 12 and 15 kDa. About 20% of patients affected by pollen allergy are sensitized against profilins. The corresponding IgE antibodies are highly cross-reactive between pollen and food profilins, but seem to have no clinical impact on food allergies [12]. The profilin of the peanut is Ara h 5 [27]. 

### The cupin superfamily

Globulin storage proteins, which account for a large share of human nutrition, belong to the superfamily of cupins. In this context, legumes, and among those particularly peanut and soybean, have been investigated most thoroughly. Globulins consist of 7S-globulins (vicilins) and 11S-globulins (legumines). Ara h 1, the allergenic peanut vicilin, is one of the main storage proteins of the peanut and is immunologically recognized by more than 90% of patients with peanut allergy. Gly m 5.01, another vicilin, is one of the most important soybean allergens. Another vicilin is Cor a 11, a hazelnut allergen. Legumines also consist of proteins with a known allergenic impact, like for example Ara h 3/4 [12]. 

### Cross-reactive carbohydrate determinants (CCD)

Allergen-specific IgE antibodies do not only recognize and bind peptide epitopes, they also specifically bind to N-glycan epitopes, so-called carbohydrate epitopes. As these are responsible for various cross-reactions, they are also called cross-reactive carbohydrate epitopes (or determinants) (CCD). They strongly limit the specificity of in vitro test systems. Only in single cases do they seem to be clinically relevant. 

Plant allergens are mainly glycoproteins and are found in pollen, particularly grass pollen, and vegetable food. Their role in peanut allergy is unclear. The only peanut allergen with a xylosylated N-Glycan is Ara h 1 [32]. Carbohydrate-specific IgE against Ara h 1 had no clinical relevance [60]. Furthermore, IgE reactivity against peanut CCDs is found in severe alcoholics, but it has no clinical impact [61]. 

## Allergens of Class I and Class II food allergies 

As a rule of thumb, allergen contact causes most severe courses in Class I food allergies, while Class II food allergies have milder courses after allergen contact. This kind of simplification can, however, be dangerous when it is not recognized that, for example, birch pollen-celery-spice allergy syndrome can lead to the most severe allergic reactions. Surprisingly, the birch pollen-associated protein of the soybean, Gly m 4 (formerly Sam 22), whose primary structure is 53% identical with Bet v 1, was identified as an allergen that can cause anaphylaxis in patients with birch pollen allergy after the ingestion of soy products [7, 31]. Thus, severe reactions due to pollen-associated food allergen sources like celery, hazelnut, or soy can also occur in adults. 

This emphasizes the necessity of identifying and characterizing the allergenic components of an allergen source. For the allergen source peanut, which primarily causes Class I food allergies, a birch pollen-profilin-homologous allergen (Ara h 5) and a Bet v 1-homologous allergen (Ara h 8) has also been detected. This suggests a pollen-associated cross-reactivity for Ara h 5 and Ara h 8. Vice versa, the allergen source hazelnut, which primarily triggers Class II allergic reactions, also contains allergens that can cause symptoms of a Class I food allergy (e.g., hazelnut lipid transfer protein (LTP) [7, 54]. 

## Matrix effects [7] 

The current problem is finding out which allergenic potency each allergen component has. So far this can only be derived indirectly. The identification of single allergens is particularly important for the diagnostic work-up with the aim to develop a customized immunotherapy for each patient. But is the search for single allergens not being overemphasized in the context of food allergies? In contrast to pollen allergy, food allergens have to be evaluated in the context of their matrix, which can vary significantly depending on the method of food preparation. Side dishes are an additional factor. In cooking, the food allergen is subject to manifold physical effects and chemical reactions, the products of which are exposed to the individual digestive process, the individual immune state, and the genetic background of each person. Antacids, for example, can trigger or increase the allergenicity of food [59]. For the major peanut allergen, Ara h 2, the reaction with sugars, the so-called Maillard reaction, has been described as increasing its allergenicity [39]. The water content of nuts and seeds can also influence the allergenicity of allergen sources [58]. In China, where peanuts are mainly used as ingredients of cooked food, few people suffer from peanut allergy, while in the USA and Europe, where peanuts are mainly eaten roasted, more people are affected by peanut allergy [9]. Another interesting observation is the fact that 11S-storage proteins tend to form aggregates, which when cooked can form gel-like structures [13]. It has to be taken into account that the digestive system is not only faced with soluble substances like antigens or allergens, but also has to interact with hardly soluble substances. In this context, it is necessary to consider the characteristics of allergens first in order to better understand the complex phenomenon of food allergy. As a paradigm we have chosen peanut allergy because it affects the highest number of patients, the triggering level of symptoms is low, and the range of clinical symptoms is wide (oral allergy syndrom to fatal anaphylactic shock). The aim of this review article is to present the structural characteristics of peanut allergens [7]. 

## Ara h 1 

Ara h 1 is a vicilin type 7S-storage protein with two conserved cupin consensus sequences and belongs to the protein superfamily of cupins. Ara h 1 is a glycoprotein with a glycosylation motif in the C-terminal area of the molecule on which a heterogeneous conglomeration of N-glycans with the structure Man5–6Glc NAc2 or Man3–4XylGlcNAc2 is conjugated through asparagine [32]. Based on the primary sequence deduced from the structural gene (accession# P43238), a molecular weight of 69 kDa without glycosylation fraction and a pI of 6.4 can be calculated. As shown by Buschmann et al. [18], Ara h 1 in peanut extract consists of two differently sized isoforms; in Western blotting, their presence is indicated by an apparent mean molecular weight of 63.5 kDa (Table 1). By 2D-electrophoresis/immunoblot, Ara h 1 can be split into at least 16 isoallergens [18]. N-terminal sequencing was able to demonstrate that the shortened form of Ara h 1 starts at amino acid 85 (G-85) and contains hydroxyproline as a post-translational modification. The calculated molecular weight without glycosylation fraction is 62 kDa. The larger form of the mature Ara h 1 (without signal peptide; starting with K-26), which can be detected using monoclonal antibodies, is obviously N-terminally blocked and thus an N-terminal analysis is impossible. Another fact that argues for the existence of a larger form of Ara h 1 is that 3 of the 23 linear IgE-reactive epitopes have been detected in the N-terminus at positions 25 – 34, 48 – 57 and 65 – 74 [7, 55]. According to unpublished results, the N-terminal fragments postulated by Wichers et al. [62] were identified using protein sequencing and location parameters in 2D electrophoresis, which would contradict the postulated N-terminal blockade (Becker, unpublished). On the other hand, in the data used in the diploma thesis of T. Latendorf [35] on peptide mass trypsin fingerprinting of intact Ara h 1, N-terminal fragments are found (interpretation by Becker, unpublished). The fame for the first description of Ara h 1 is attributed to Burks et al. [17], although the authors could not demonstrate the Con A-reactivity of the allergenic glycoprotein that Barnett and Howden [4] had described as early as 1986 and that was identical in all other characteristics. In 1996, Buschmann et al. [18] demonstrated the Con A-reactivity of Ara h 1. Burks et al. [17] were, however, the investigators who registered Ara h 1 with the IUIS Allergen Nomenclature Subcommittee. Ara h 1 tends to form stable homotrimers against proteolysis by hydrophobic interaction [40] which, in case of depletion, form stable IgE-fragments, for example at approximately 35 kDa in Western blot [7]. 

## Ara h 3, Ara h 4 [7] 

**Figure 1.**
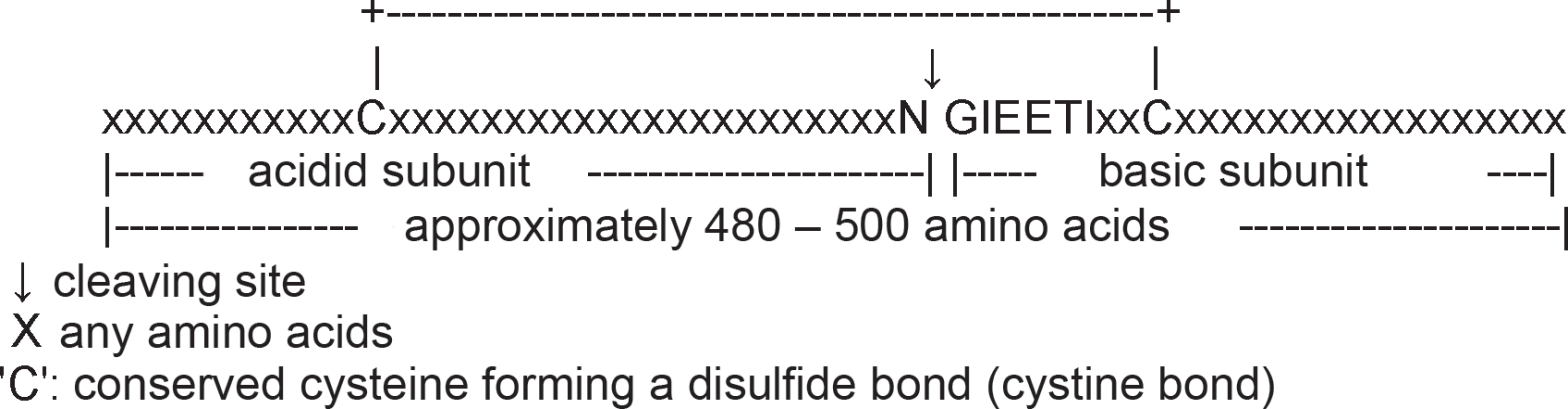
Principal structure of 11S proteins like Ara h 3 and Ara h 4 with post-translational interface and cystine positions (IPR006044 → http://www.ebi.ac.uk/interpro/DisplayIproEntry?ac=IPR006044) [7].

The allergens Ara h 3 and Ara h 4 are isoallergens with a sequence identity of the deduced primary structure of almost 92%. The subcommittee is to blame for the chaotic nomenclature [5] as they accepted an unauthorized naming, namely “Ara h 3”, for the orginal 14-kDa fragment [15, 23]. Ara h 3/4 are legumin type 11S-storage proteins and possess two conserved cupin domains. They therefore also belong to the cupin protein superfamily. The theoretically calculated molecular weights of the deduced primary structures of the structure genese Ara h 3 and Ara h 4 are 58.8 kDa with a pI of 5.8. As typical for an 11S-signature, the proteins are post-translationally split into an acidic and a basic fragment; this results in a more compact storage within the peanut. In a first approach one could say they are heterodimers (Figure 1). This is supported by Western blotting under reducing conditions where the acidic fragment breaks down to further subunits. Interestingly, for the acidic recombinantly produced subunit trypsin, inhibitory properties have been demonstrated in experiments [22]. It can be assumed that this contributes to the allergen’s resistance to digestion and thus to its allergenicity. In the acidic fragment of Ara h 3, all 4 of the linear epitopes detected so far have been found; IgE-reactivity could also be shown for the basic fragment [10, 49]. Under native conditions, Ara h 3/4 (hexamers) and Ara h 1 (trimers) build aggregates or are extracted as aggregates. Superdex 200 gel permeation chromatography demonstrated that these aggregates have a molecular weight of more than 200 kDa in the exclusion volume and account for approximately 90% of proteins of the peanut extraction. The detailed analysis of the Ara h 3/4 complex using 2D electrophoresis, immunoblot and peptide mass trypsin fingerprinting led to the detection of at least 4 isoallergens and the identification of their acidic and basic subunits [10]. 

## Ara h 2, Ara h 6, and Ara h 7 

Ara h 2 is a major peanut allergen. It is a conglutin type 2S-storage protein and, similar to Ara h 6 and Ara h 7, belongs to the protein superfamily of prolamins. In peanut extract, Ara h 2 is present in two isoforms, the molecular weight of which differs by 1,432 Da, corresponding to a peptide insertion of 12 amino acids [20]. This insertion could be deduced from the corresponding Ara h 2 structure genes [20]. In 2D electrophoresis/immunoblot of the peanut extract, both isoforms have at least 3 isoallergens [51]. The molecular weights of the isoforms were determined using Matrix Assisted Laser Desorption/Ionisation, Time Of Flight (MALDI-TOF) correspond well with the molecular weights than can be deduced from the structure genes. This suggests that, contrary to earlier publications [16], Ara h 2 is not glycosylated. This is supported by mass-spectrometric measurements carried out by Li et al. [38]. These authors could additionally show that some prolines are hydroxylated and that the isoforms are formed by a lack of amino acids in the C-terminus or by exchanges of amino acids in the primary sequence. 

This is interesting as Ara h 2 has an N-glycolylation motif. Due to the fact that Ara h 2 belongs to the protein superfamily of prolamins, it can be assumed that the biological function of Ara h 2 is to inhibit amylase or trypsin. Indeed a trypsin inhibitory activity could be demonstrated in experiments [41]. This could possibly explain the strong resistance against digestion. When peanut products are used for baking, Ara h 2 is the most stable of all peanut allergens [24]. In Ara h 2, the Maillard reaction results in an increase of IgE-reactivity [39]. This could be due to the fact that among the 10 detected IgE-reactive epitopes some are better presented by the Maillard reaction with increaing affinity. The formation of neoallergens as an alternative explanation could not be verified so far [7]. 

The primary sequences of Ara h 6 and Ara h 2 are 53% identical. The difference between Ara h 6 and Ara h 2 is that Ara h 6 has 10 cysteins instead of 8 and does not have an N-glycosylation motif. Ara h 6 is resistant to digestion and stable to heat, i.e., in baking processes [57]. The three-dimensional structure of Ara h 6 (1W2Q) has been described completely [37]. In 2D electrophoresis/immunoblot at least 4 isoallergens can be identified for Ara h 6. In the extract, a C-terminal fragment starting with the amino acid S-48, which is IgE-reactive, has been found. An N-terminal fragment at approximately 6 kDa is not IgE-reactive (see also the results of Marsh et al. [42]). This supports the suggestion that Ara h 6 possesses conformational epitopes, particularly at the C-terminal end of the molecule, and that a cross-reactivity to Ara h 2 exists via conformational epitopes. This raises the question whether IgE-reactivities against Ara h 6 are exclusively acquired via cross-reactivity with Ara h 2, or if an independent sensitization is present. As some patients with peanut allergy react only to Ara h 2 or Ara h 6 in Western blot, it has to be assumed that an indpendent sensitization through Ara h 6 is possible [7]. Interestingly, Ara h 2 and Ara h have the greatest effector activity within the peanut extract [46]. 

**Figure 2.**
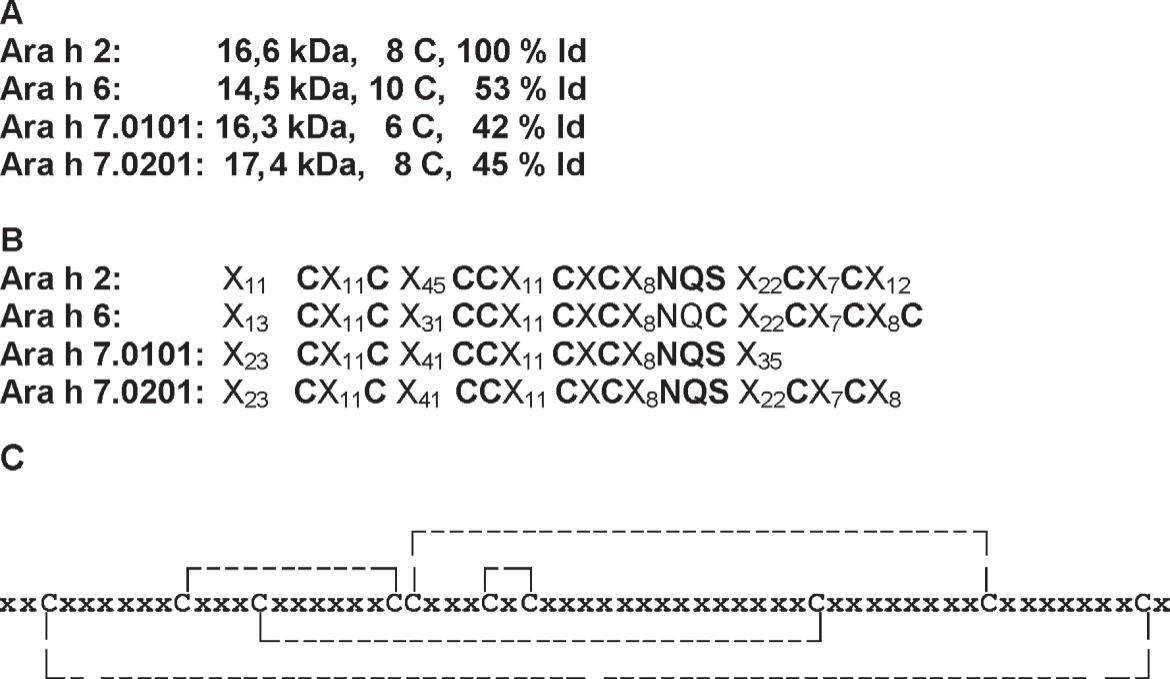
Structural characteristics of allergenic peanut conglutines. A: Calculated molecular weight of mature allergens, number of cysteins C and percentage of identic amino acids in the primary sequence in relation to Ara h 2. B: Positions of cysteins C and the glycosylation signal sequence NXS/F in the primary sequence of conglutins. C: Suggested position of the – S-S links of cystines (IPR006106 → http://www.ebi.ac.uk/interpro/Dis playIproEntry?ac=IPR006106) [8].

Due to its IgE-reactivity Ara h 7 has been found, cloned, and recombinantly produced via the phage display system [27]. Ara h 7 could not be identified in the peanut extract at first. Its primary amino acid sequence identity with Ara h 2 is 42% [7, 8]. It possesses only 6 cysteins but has an N-glycosylation motif. Schmidt et al. [52] managed to clone an Ara h 7 isoform with 8 cysteins. This isoform was registered by the IUIS Nomenclature Subcommittee as Ara h 7.0201 (Table 1). In a proteome analysis of peanut extract with proteins enriched below 20 kDa, Ara h 7 could be identified. Using peptide mass trypsin fingerprinting, Schmidt et al. [52] managed to verfy this isoform as an authentic allergen and identified further isoforms. An overview of the structural differences between the 4 conglutines is presented in Figure 2 [8, modified]. 

## Ara h 5 and Ara h 8 

**Figure 3.**
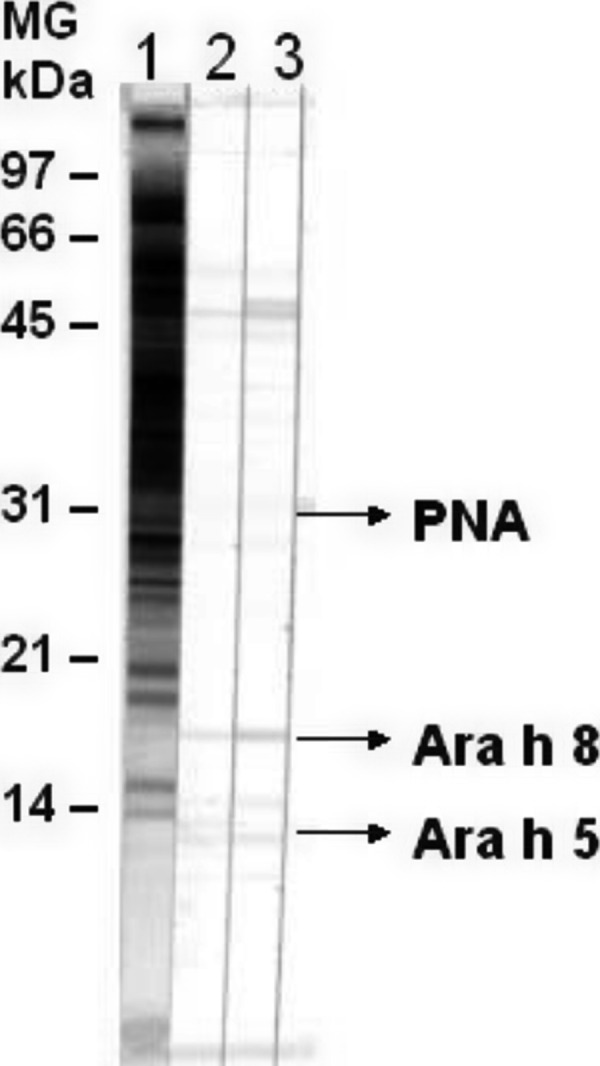
Western blot of peanut extracts for better visibility of Ara h 5 and Ara h 8 extracted at pH 5. 1: Protein staining (India ink); 2 and 3: IgE reactivity of patients with peanut and birch pollen allergy [8, modified].

For Ara h 5, peanut profilin, and Ara h 8, a Bet v 1-homologuous allergen, it is suggested that the IgE-reactivity is based on cross-reactivity with an airborne allergen. As a DBPCFC (double-blind placebo-controlled food challenge) with recombinant single allergens represents a complex problem and is not possible to date, conclusions on allergenicity and clinical relevance of the individual allergens can only be drawn indirectly. Ara h 5 and Ara h 8 cannot be detected as proteins in Western blot on nitrocellulose with India ink nor on PVDF membrane with Coomassie blue; they can however be demonstrated immunologically (Figure 3) [7]. Thus, Ara h 5 and Ara h 8 should only be present in peanut extract in low concentrations. Ara h 5 has a sequence idenitity of 72% with birch pollen profilin [28]. Interestingly, in Western blot inhibiton testing, rBet v 2 is not able to inhibit the binding to Ara h 5. The vice versa of this experiment, on the other hand, is successful. 

These findings are confirmed by homology modeling and epitope analysis, with Ara h 5 presenting a specific, i.e., non-crossreacting epitope [19]. 

This single finding questions Bet v 2 as a primary source of sensitization; this would have to be confirmed by a larger series of patient sera [7]. An Ara h 8 study [44], in which only 30% of patients were sensitized against Bet v 2, shows that in patients with birch pollen allergy, Ara h 5 is only of minor importance for the question of cross-reactivity with peanut. In patients with birch pollen allergy, Ara h 8 is a major allergen [7], which can be misinterpreted as peanut allergy due to serologic cross-reactivity with Bet v 1. When the IgE levels against birch pollen extract are higher than those against peanut extract, only mild symptoms against peanut can be present [3]. Ara h 8 is extremely susceptible to digestion; in roasting processes, it loses 9 times more IgE reactivity when untreated peanuts are compared to roasted peanuts usually sold in their shells [7, 44]. Riecken et al. [50] managed to clone an isoform of Ara h 8, Ara h 8.0201, (Table 1) and demonstrate its authenticity in peanut extract via peptide mass trypsin fingerprinting [8]. 

As for the major birch pollen allergen Bet v 1, a hydrophobic pocket for the homologous peanut allergen Ara h 8 could be demonstrated experimentally; in natural Ara h 8 it was, however, loaded with lipids [48]. According to first experiments, the binding of lipids to the allergen seems to protect the latter from gastric digestion [48]. 

## Peanut hemagglutinin [7] 

Peanut hemagglutinin, better known as PNA, is not registered as an allergen with the IUIS Allergen Nomenclature Subcommittee, but has been described by Burks et al. [14] as a minor allergen. It is a tetramer and its basic unit has a molecular weight of 30 kDa. In Western blot it shows IgE reactivity with 4 of 70 sera from patients with peanut allergy (Becker, unpublished) and is therefore of minor importance as a potential allergen. PNA is commercially available but immunologically contaminated with other peanut allergens [7]. 

## Ara h 9, the peanut LTP 

In 2002, the presence of peanut LTP in peanut extract was described indirectly for the first time [2]. In the usual peanut extracts that are extracted with slightly basic buffers, Ara h 9 could not be detected in the 9 kDa area. Only in extracts produced with acidic buffers could Ara h 9 be detected. Two isoforms of Ara h 9 (Table 1) could be cloned, expressed, and registered with the IUIS Allergen Nomenclature Subcommittee [8, 34]. For patients from Mediteranean countries, Ara h 9 has a clinical impact [34, 36]. It has not been fully ilucidated yet as to whether sensitization takes place via cross-reactivity with peach LTP (Pru p 3) or also independently via peanut. These patients with peanut allergy do not exhibit the classic IgE reactivities agains Ara h 1, Ara h 2, or Ara h 3/4.This emphasizes the influence of allergen-related sensitization patterns (allergograms) through the geographically determined flora. 

## Ara h 10, Ara h 11, and a further peanut oleosin [8] 

Ara h 10 and Ara h 11 have been registered as allergenic peanut oleosins with the IUIS Allergen Nomenclature Subcommittee, but have not been published yet (Table 1) (May 2011). The first peanut oleosin has been described by Pons et al. [45] as a minor allergen, but could not be registered as an allergen with the IUIS Allergen Nomenclature Subcommittee. 

The monomer has a molecular weight of approximately 18 kDa. It also exists in dimeric and further aggregate forms. Its role as an allergen remains unclear [45]. 

## The peanut allergome 

**Figure 4.**
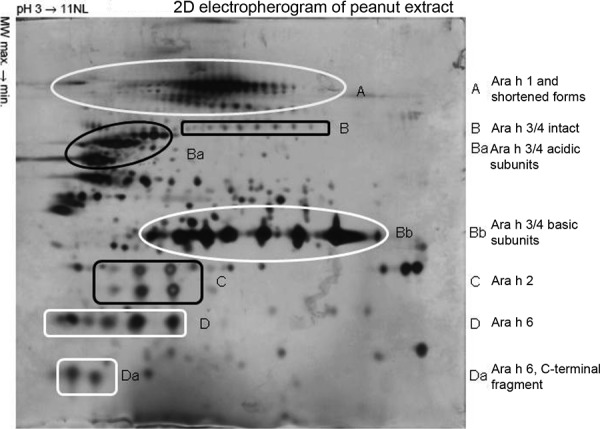
2D electropherogram of peanut extract: From [28] (Figure 4a), modified. Copyright: Wiley-VCH Verlag GmbH & Co. KGaA. Reproduced with permission. Allocation of allergens according to Figure 3 in [41]. First dimension IEF (pH area 3 – 11, non-linear, 18 cm). Second dimension 14% SDS-PAGE under reducing conditions. Proteins were made visible using silver staining.

The peanut allergome includes the IgE-reactive proteins and glycoproteins of the peanut proteome (Figure 4). The molecular visual presentation of allergens is mainly based on Western blotting where the proteins of the allergen source are separated in the SDS-PAGE, transferred to a transfer medium (nitrocellulose or PVDF), and the IgE-reactive bands (allergens) are made visible. This sufficed to molecularly identify the 11 peanut allergens and contribute to the elucidation of their structure. Nevertheless, the value of Western blotting is limited, as will be shown in two examples. As Western blot contains protein-denaturing stepts, the presence of allergens under physiologic conditions is not always correctly described. Ara h 1, for example, is present as a trimer (~ 210 kDa) under physiologic conditions, which can be indirectly deduced from a combination of size exclusion chromatoghraphy and Western blot technique. Also, the presence of isoallergens is not sufficiently displayed by Western blot. This problem can be solved by two-dimensional gel electrophoresis with isoelectric focusing in the first dimension and SDS-PAGE in the second dimension with subsequent immunoblot. With the development of 2D electrophoresis, an essential precondition for proteom analysis has been created. The proteom of an organism is the entire set of expressed proteins in relation to predefined general conditions such as basic material and extracting conditions, which are then displayed in the 2D electropherogram (Figure 4) [6]. The analysis is carried out half-automatically in a high-throughput procedure using the peptide mass fingerprint procedure; the spots are punched out and the obtained proteins are fragmented enzymatically (e.g., trypsin) and transferred to a spectrometer for determination of their masses. For detection, MALDI-TOF mass spectrometry or electrospray ionization tandem mass spectrometry (ESI-MS/MS) is used. Subsequently, the masses of protein-specific fragments are compared with those from a data base. This means that only known proteins can be identified. MS/MS measurements can, however, also determine sequences of unknown proteins [6]. 

Natural Ara h 7 was successfully identified by proteom analysis of a peanut protein fraction below 20 kDa [52]. Two-dimensional difference gel electrophoresis (2D-DIGE) is closely related to proteome analysis. In 2D-DIGE, up to three samples can be marked with different fluorescence stains and simultaneously examined in an electrophoresis run. Additive color mixing shows which proteins of the compared samples dominate, are present in the same amounts, or are underrepresented [51]. By combining proteomics with immunoblotting, allergen analysis reaches another dimension that could lead to the description of another allergen source’s allergome. This technique will become more and more important for the standardization of allergen extracts and allergen products [53]. 

While diagnostic work-up with single allergens is currently of minor importance for animal-derived food, several marker allergens are available for food allergen sources from vegetable food [29, 30]. The following single allergens are suspected to be risk molecules for systemic reactions: peanut storage protein Ara h 2, peanut lipid transfer protein (Ara h 9), hazelnut LTP Cor a 8, peach LTP Pru p 3, and wheat protein omega-5-gliadin Tri a 19 [30]. 

All in all, IgE detection based on single allergens provieds a molecule-specific diagnostic work-up; its impact remains to be elucidated according to allergen source and clinical characterization of the single allergens. 

## Allergen-poor peanuts 

An idea favored in the USA to solve the problem of peanut allergy is the genetic development of allergen-poor peanuts [21]. The identification of 11 peanut allergens, which can be assumed to be final, provides a prerequisite for this. Given that the major allergens Ara h 1 and Ara h 3/4 as storage proteins make up ~ 90% of all peanut proteins, the question arises as to what would remain of a peanut were these allergens reduced or removed by genetic engineering. We had the opportunity to examine a naturally Ara h 1-deficient variety from Indonesia. The proteome analysis showed the absence of Ara h 1 and a reduction of Ara h 2 as compared to the standard peanut [51]. Immunologic investigation confirmed that the Ara h 1 content of the Indonesian variety was significantly reduced. RBL testing (mast cell degranulation test) did, however, not show a reduced allergenicity of this variety as compared to the standard peanut [33]. Even though the functional examination of the Ara h 1-deficient peanut variety has to be classified as preliminary, it shows the complexity of this approach, particularly as the immune system’s reaction to “allergen-poor” peanuts cannot be predicted. 

## Summary 

The identification and characterization of relevant single allergens, their cloning and production as recombinant allergenic proteins, as well as the synthesis of IgE epitope representing peptides has allowed for a component-resolved diagnosis (CRD) by making possible the detection and quantifation of IgE antibodies that are specific for an allergenic protein or even for a sequential epitope. This way, patients’ individual sensitization patterns against various proteins (e.g., allergenic food, pollen, and so on), or homologous proteins in different types of food, or various epitopes of a single allergen molecule can be demonstrated. 

CRD only makes sense when it has therapeutic consequences now or in the future [7]. As currenlty there is no safe and efficacious allegen-specific immunotherapy with peanut extracts for patients with peanut allergy, the identification of all peanut allergens is the basis for the development of a safe, customized immunotherapy or for the development of allergen-poor peanuts [7]. From the structural data, it can be concluded to what extent allergens can interact with the matrix, and whether neoallergens are possible. Based on the single allergens, their interaction with the gastrointestinal tract and the immune system can be put on a molecular foundation [7]. Thus, peanut allergens play a key role in the further develompent of diagnosis and immunotherapeutics as well as in the elucidation of the pathomechanisms of food allergy and finally also in prevention [7]. A summary of the structural data of peanut allergens is presented in Table 1. Figure 3 shows the positions of the difficult to detect allergens Ara h 8, Ara h 5, and PNA in Western blot under reduction conditions [7], and Figure 4 shows the allergens Ara h 1, Ara h 2, Ara h 3/4, and Ara h 6 with their isoallergens in 2D electrophoresis. 

## Annotation 

This review article is a comprehensive update of the following review articles: 

*Becker WM, Schocker F, Boldt A.* Allergene der Erdnuss: Struktur und Charakteristika. Allergologie. 2005; *28:* 359-366 [7]. 

*Becker WM, Schocker F, Petersen A.* Allergene der Erdnuss: Struktur und Charakteristika. In: Werfel T, Wüthrich B. (Hrsg). Nahrungsmittel und Allergie 3. München – Orlando: Dustri; 2010, 156-66 [8]. 

**Table 1.** Characteristics of peanut allergens [8, modified].

**Allergen**	**MGber (kDA)**	**pI**	**Structural characteristics**	**N-Terminus**	**Protein family**	**Function**	**Accession#**
Ara h 1	68.757	6.36	Glycoprotein	K26SSPYQK	7S globulin	Storage	P43238
	61.882	6.03	Glycoprotein shortened isoform	G85SpPGE	Vicilin	Protein	
							
Ara h 2	17.993	5.51		R22QQEL	2S Albumin	Trypsin	AY158467
	16.637	5.5		R22QQEL	Conglutin	Inhibitor	L77197
							
Ara h 3	58.697	5.58		I21SFRQQ	11S Globulin	Storage	ACC63045
Ara h 3 iso	56.241	5.35	Isoallergens forms hexamer	V19FTRQGG	Legumin	proteins	AAT39430
Ara h 4	58.849	5.47		I21SFRQQ			AF086821
Ara h 3/4*	23.115	6.58	Trypsin inhibitor rec.	I23SFRQQ			AF487543
							
Ara h 5	14.051	4.58	Profilin	MSWQTYV	Profilin		AF059615
							
Ara h 6	14.491	5.02		M21RRERGR	Conglutin		AF092846
							
Ara h 7.0101	16.322	5.56		T21RWDPD	Conglutin		AF091737
Ara h 7.0201	17.374	7.49		T21RWDPD			EU046325
							
Ara h 8.0101	16.952	5.03	Bet v 1 homologue	MGVFTF		PR10-	AY328088
Ara h 8.0201	16.281	5.07	Bet v 1 homologue	GVHTFEE		Protein	EF436550
							
Ara h 9.0101	9.135	9.45		I25SCGQVN		nsLTP	EU159429
Ara h 9.0201	9.054	9.25		L25SCGQVN			EU161278
							
Ara h 10.0101	15595	9.36		MTDRTQP		Oleosin	AY722694
Ara h 10.0102	17752	9.61		MTDRTQP			AY722695
							
Ara h 11.0101	14307	9.08		(M)AEALYY		Oleosin	DQ097716
							
Oleosin	18.435	9.8		MATATDRA		Oleosin	AF325917
							
PNA	26.749	5.03	Exists as tetramer	A24ETVSFN		Lectin	S42352
